# Supplementary material for: A Higher radix architecture for quantum carry-lookahead adder
Source: Sci Rep. 2023 Sep 28;13:16338. doi: 10.1038/s41598-023-41122-4 (PMC10539406; doi:10.1038/s41598-023-41122-4)
Supplement: Supplementary file 1 — Supplementary Information. [file 41598_2023_41122_MOESM1_ESM.pdf]

Supplementary Material
A Higher Radix Architecture for Quantum Carry-lookahead Adder

Siyi Wang[*,1], Anubhab Baksi[*,2], and Anupam Chattopadhyay[*,3]

[*]School of Computer Science and Engineering, Nanyang Technological University, Singapore, 639798
[1]siyi002@e.ntu.edu.sg
[2]anubhab.baksi@ntu.edu.sg
[3]anupam@ntu.edu.sg

A Worked-out Examples

Here, we use several specific numerical examples to show the workflow of the higher radix adder.

We assume that the binary number a is 101001, and b is 010011. Additionally, the first carry bit c_0 is set to 0. Our target is to get the sum s of a and b. For this specific six-bit addition, when the radix is changed from from 6 to 1, the calculation process is as follows.

- $\mathbf{r = 6}$.

  When the radix is equal to the bit width of the binary addends, our higher radix adder is just a simple ripple carry adder that only calculates the sum path. The calculation process of the sum path is as follows.

Sum path:

	a	101001
+	b	010011
	s	111100

Calculate p:

	a	101001
$\oplus$	b	010011
	p	111010

Calculate g:

	a	101001
$\times$	b	010011
	g	000001

For the remaining adders, most of them need to go through four steps, p and g calculation, higher radix structure, carry path, and sum path. The first step, calculating p and g, is the same for all the remaining examples. The calculation process is shown above.

After obtaining p and g, the remaining steps differ for the higher radix adder with different radix. The details of the calculation of our higher radix adder with its radix set from 5 to 1 are shown below.

- $\mathbf{r = 5}$. To begin with, since we do not need to compute the carry of the most significant bit, the most significant p and g are ignored. Firstly, the higher radix structure divides the remaining p and g into groups of five each, yielding a p_{group} and a g_{group}. In the carry path, according to the Brent-Kung structure, we do not need any operation. In this case, c_5 and g_{group} are equal, both being 0. In the sum path, the ripple carry structure is used to add a, b; and the computed carry c by the group to obtain the sum s.

  Higher radix structure:

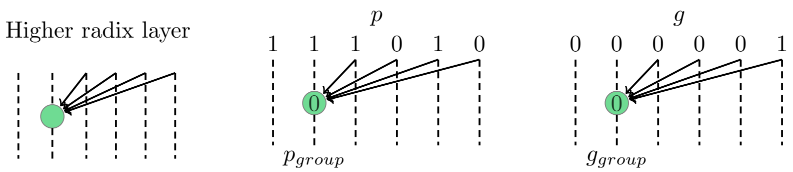

  Carry path:

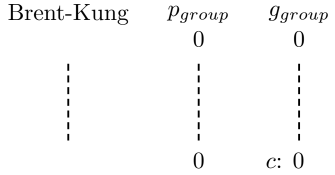

Sum path:

a	101001	
b	010011	
c	0	0
s	111100	

- $\mathbf{r = 4}$. Similar to radix 5, we show radix-4 addition below.

  Higher radix structure:

  Carry path:

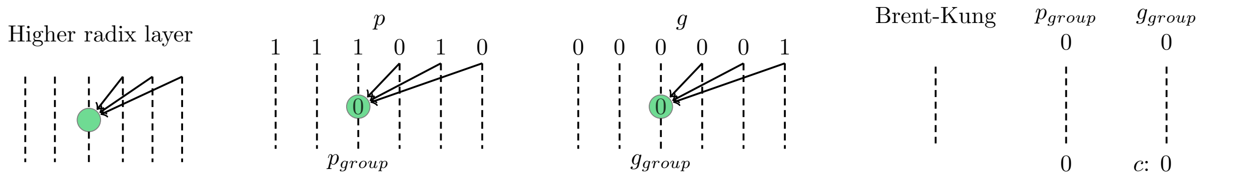

Sum path:

a	101001
b	010011
c	0 0
s	111100

- **r = 3**. Similarly, the following is the process for an adder with radix 3.

  Higher radix structure: Carry path:

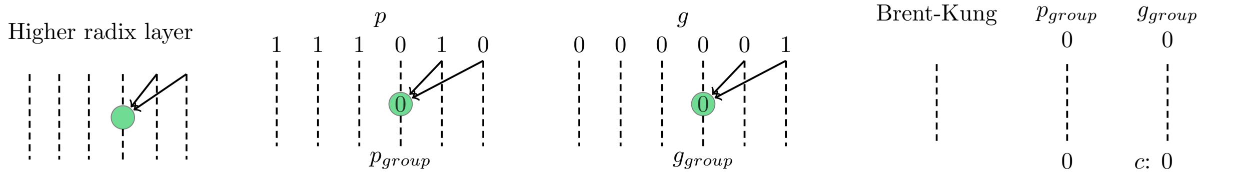

Sum path:

a	101001
b	010011
c	0 0
s	111100

- **r = 2**. The overall calculation process is shown as follows.

  Higher radix structure: Carry path:

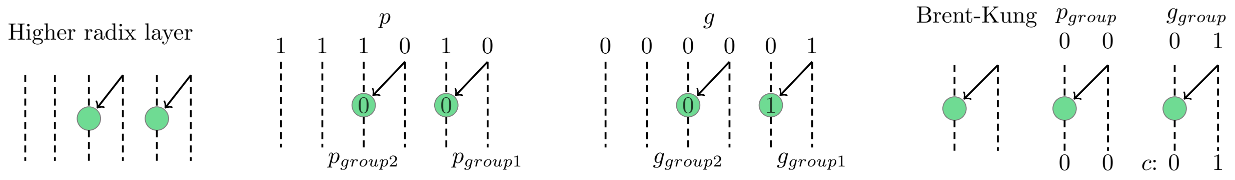

Sum path:

a	101001
b	010011
c	0 1 0
s	111100

- **r = 1**. When the radix is one, it is a special case. Since the higher radix structure does not work, it is essentially a CLA.

  Carry path:

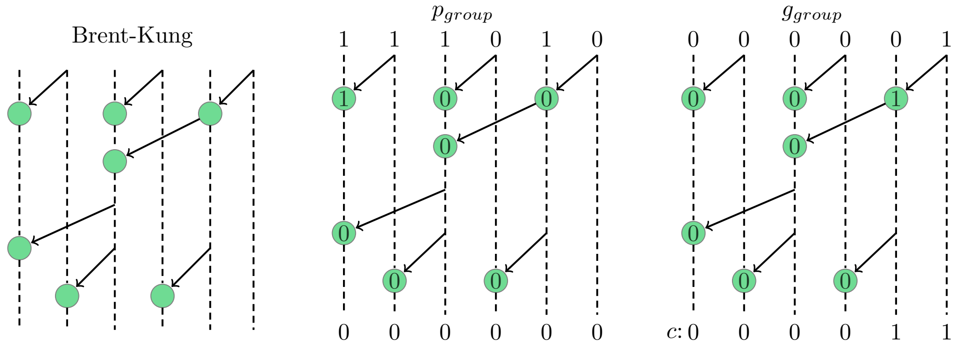

Sum path:

a	101001
b	010011
c	000110
s	111100

B Derivation Details of Cost Formulae

Here, we show the details of adding the sub-cost formulae of the seven steps to obtain the final cost formulae in section 3.5.In Derivations (14), (15), (16) respectively; we show the step-by-step derivation for the T-count and T-depth.

T-count

$$\begin{aligned}
&=\overbrace{7(n-\alpha)}^{\text{Step 1}}+\overbrace{\rho[7+4(2r-3)]}^{\text{Step 2}}+\overbrace{2\cdot 7[2\rho-1-\omega(\rho)-\lfloor\log(\rho)\rfloor]}^{\text{Step 3}}+\overbrace{7[2\rho-1-\omega(\rho)-\lfloor\log(\rho)\rfloor]}^{\text{Step 4}}+\overbrace{0}^{\text{Step 5}}+\overbrace{7\rho}^{\text{Step 6}}+\overbrace{4(n-\lceil\frac{n}{r}\rceil)}^{\text{Step 7}}\\
&=7n-7\alpha+7\rho+8r\rho-12\rho+28\rho-14-14\omega(\rho)+14\rho-14\lfloor\log(\rho)\rfloor-7-7\omega(\rho)-7\lfloor\log(\rho)\rfloor+7\rho+4n-4\lceil\frac{n}{r}\rceil\\
&=44\rho-4(\lceil\frac{n}{r}\rceil-1)-4+8r\rho+11n-7\alpha-21-21\omega(\rho)-21\lfloor\log(\rho)\rfloor\\
&=40\rho+8r\rho+11n-7\alpha-25-21\omega(\rho)-21\lfloor\log(\rho)\rfloor\\
&=(8r+40)(\lceil\frac{n}{r}\rceil-1)+11n-25-7[(n-1)\pmod r+1]-21\omega(\lceil\frac{n}{r}\rceil-1)-21\lfloor\log(\lceil\frac{n}{r}\rceil-1)\rfloor\\
&=(8r+40)(\lceil\frac{n}{r}\rceil)+11n-7-25-40-8r-7\cdot(n-1)\pmod r-21\omega(\lceil\frac{n}{r}\rceil-1)-21\lfloor\log(\lceil\frac{n}{r}\rceil-1)\rfloor\\
&=(8r+40)\cdot\left\lceil\frac{n}{r}\right\rceil+11n-72-8r-7\cdot(n-1)\pmod r-21\omega(\left\lceil\frac{n}{r}\right\rceil-1)-21\left\lfloor\log(\left\lceil\frac{n}{r}\right\rceil-1)\right\rfloor)
\end{aligned} \tag{1}$$

T-depth

$$\begin{aligned}
&=\overbrace{3}^{\text{Step 1}}+\overbrace{[3+r-1+\beta]}^{\text{Step 2}}+\overbrace{3\cdot(\lfloor\log(\rho)\rfloor+\left\lfloor\log\frac{\rho}{3}\right\rfloor+2)}^{\text{Step 3}}+\overbrace{3\cdot(\lfloor\log\rho\rfloor+\left\lfloor\log\frac{\rho}{3}\right\rfloor+1)}^{\text{Step 4}}+\overbrace{0}^{\text{Step 5}}+\overbrace{3}^{\text{Step 6}}+\overbrace{r}^{\text{Step 7}}\\
&=6(\lfloor\log(\rho)\rfloor+\left\lfloor\log\frac{\rho}{3}\right\rfloor)+17+2r+\beta\\
&=6(\lfloor\log(\rho)\rfloor+\left\lfloor\log\frac{\rho}{3}\right\rfloor+4)+17+2-24+\lfloor\log(r-2)\rfloor+2r\\
&=6\cdot(\left\lfloor\log(\left\lceil\frac{n}{r}\right\rceil-1)\right\rfloor+\left\lfloor\log(\frac{1}{3}(\left\lceil\frac{n}{r}\right\rceil-1))\right\rfloor+4)+\lfloor\log(r-2)\rfloor+2r-5
\end{aligned} \tag{2}$$

QC

$$\begin{aligned}
&=\overbrace{[3\cdot n-\alpha]}^{\text{Step 1}}+\overbrace{[\rho\cdot(r-1)]}^{\text{Step 2}}+\overbrace{[2\cdot\rho-1-\omega(\rho)-\lfloor\log(\rho)\rfloor]}^{\text{Step 3}}+\overbrace{0}^{\text{Step 4}}+\overbrace{0}^{\text{Step 5}}+\overbrace{0}^{\text{Step 6}}+\overbrace{[\alpha-1+(r-2)\rho]}^{\text{Step 7}}\\
&=3n-\alpha+r\rho-\rho+2\rho-1-\omega(\rho)-\lfloor\log(\rho)\rfloor+\alpha-1+r\rho-2\rho\\
&=3n+(2r-1)\rho-2-\omega(\rho)-\lfloor\log(\rho)\rfloor\\
&=3n+(2r-1)(\lceil\frac{n}{r}\rceil-1)-2-\omega(\lceil\frac{n}{r}\rceil-1)-\lfloor\log(\lceil\frac{n}{r}\rceil-1)\rfloor\\
&=3n+1-2-2r-\omega(\lceil\frac{n}{r}\rceil-1)+(2r-1)\lceil\frac{n}{r}\rceil-\lfloor\log(\lceil\frac{n}{r}\rceil-1)\rfloor\\
&=3n-1-2r-\omega(\left\lceil\frac{n}{r}\right\rceil-1)+(2r-1)\cdot\left\lceil\frac{n}{r}\right\rceil-\left\lfloor\log(\left\lceil\frac{n}{r}\right\rceil-1)\right\rfloor
\end{aligned} \tag{3}$$

C Optimum Radix

According to the cost formulae in Table 4, here we will analyze how to find the best radix in the case of large input size in detail. First, we divide the radix into three cases: small, medium and large, and use r_s, r_m and r_l to denote them respectively. Furthermore, n is used to denote input size and r is used to denote the radix. It is assumed that $O(r)$ is equal to $O(1)$ in the r_s case, $O(r)$ is equal to $O(\sqrt{n})$ in the r_m case, and $O(r)$ is equal to $O(n)$ in the r_l case. Besides, r should be strictly less than or equal to n.

For our adder which is denoted by $\star$, the asymptotic complexity of T-count, T-depth and QC is shown below.

$$r_s \begin{cases} T-count & = 56n - 7\frac{n}{r}; \\ T-depth & = 12\log n; \\ QC & = 4n + \frac{n}{r} \end{cases} r_m \begin{cases} T-count & = 56n; \\ T-depth & = 12\log n + 9r; \\ QC & = 4n \end{cases} r_l \begin{cases} T-count & = 56n - 7(n-1) \pmod r; \\ T-count & \geq 56n - 7r; \\ T-depth & = 9r; \\ QC & = 4n \end{cases} \quad (4)$$

For T-depth, the best radix is in the range of r_s or r_l. For T-count, the best radix is in the r_s range. For QC, the best radix is in the range of r_l.

For our adder which is denoted by $\bullet$, the asymptotic complexity of T-count, T-depth and QC is shown below.

$$r_s \begin{cases} T-count & = 46n + 3\frac{n}{r}; \\ T-depth & = 12\log n; \\ QC & = 4n + \frac{n}{r} \end{cases} r_m \begin{cases} T-count & = 46n; \\ T-depth & = 12\log n + 7r; \\ QC & = 4n \end{cases} r_l \begin{cases} T-count & = 46n - 7(n-1) \pmod r; \\ T-count & \geq 46n - 7r; \\ T-depth & = 7r; \\ QC & = 4n \end{cases} \quad (5)$$

For T-depth, the best radix is in the range of r_l. For T-count, the best radix is in the r_s range. For QC, the best radix is in the range of r_l.

For our adder which is denoted by $\diamond$, the asymptotic complexity of T-count, T-depth and QC is shown below.

$$r_s \begin{cases} T-count & = 19n + 40\frac{n}{r}; \\ T-depth & = 12\log n; \\ QC & = 5n - \frac{n}{r} \end{cases} r_m \begin{cases} T-count & = 19n; \\ T-depth & = 12\log n + 2r; \\ QC & = 5n \end{cases} r_l \begin{cases} T-count & = 19n - 7(n-1) \pmod r; \\ T-count & \geq 19n - 7r; \\ T-depth & = 2r; \\ QC & = 5n \end{cases} \quad (6)$$

For T-depth, the best radix is in the range of r_l. For T-count, the best radix is in the r_s range. For QC, the best radix is in the range of r_s.

D Comparison tables of the cost required by different quantum adders

Table 1 and table 2 show the specific data of Figures 13 and 14, respectively.

References

1. Vedral, V., Barenco, A. & Ekert, A. Quantum networks for elementary arithmetic operations. *Phys. Rev. A* **54**, DOI: 10.1103/PhysRevA.54.147 (1995).
2. Cuccaro, S. A., Draper, T. G., Kutin, S. A. & Moulton, D. P. A new quantum ripple-carry addition circuit (2004). quant-ph/0410184.
3. Draper, T. G., Kutin, S. A., Rains, E. M. & Svore, K. M. A logarithmic-depth quantum carry-lookahead adder (2004). quant-ph/0406142.
4. Takahashi, Y. & Kunihiro, N. A fast quantum circuit for addition with few qubits. *Quantum Inf. & Comput.* **8**, 636–649, DOI: 10.26421/QIC8.6-7-5 (2008).
5. Takahashi, Y., Tani, S. & Kunihiro, N. Quantum addition circuits and unbounded fan-out (2009). 0910.2530.
6. Gidney, C. Halving the cost of quantum addition. *Quantum* **2**, 74, DOI: 10.22331/q-2018-06-18-74 (2018).

Table 1. Comparison of the cost required by different quantum adders.

(a) T-count Comparison

Structure \ input size	16	32	64	128	256	512	1024
⋆ VBE RCA[1]	434	882	1778	3570	7154	14322	28658
⋄ VBE RCA[1]	134	262	518	1030	2054	4102	8198
⋆ Cuccaro RCA[2]	217	441	889	1785	3577	7161	14329
⋄ Cuccaro RCA[2]	67	131	259	515	1027	2051	4099
⋆ Draper In-place CLA[3]	819	1876	4053	8470	17367	35224	71001
⋄ Draper In-place CLA[3]	559	1306	2853	6000	12347	25094	50641
⋆ Takahashi Adder[4]	3136	6272	12544	25088	50176	100352	200704
⋆ Takahashi RCA[5]	217	441	889	1785	3577	7161	14329
⋄ Takahashi RCA[5]	67	131	259	515	1027	2051	4099
⋆ Takahashi Combine Adder[5]	784	1568	3136	6272	12544	25088	50176
⋄ Gidney RCA[6]	60	124	252	508	1020	2044	4092
⋆ Our Adder	539	1281	2954	6503	13454	27132	54607
• Our Adder	357	991	2364	5323	11283	23149	46683
⋄ Our Adder	178	471	1108	2497	5317	10876	21923

(b) T-depth Comparison

Structure \ input size	16	32	64	128	256	512	1024
⋆ VBE RCA[1]	186	378	762	1530	3066	6138	12282
⋄ VBE RCA[1]	52	100	196	388	772	1540	3076
⋆ Cuccaro RCA[2]	93	189	381	765	1533	3069	6141
⋄ Cuccaro RCA[2]	18	34	66	130	258	514	1026
⋆ Draper In-place CLA[3]	57	69	81	93	105	117	129
⋄ Draper In-place CLA[3]	48	60	72	84	96	108	120
⋆ Takahashi Adder[4]	360	450	540	630	720	810	900
⋆ Takahashi RCA[5]	93	189	381	765	1533	3069	6141
⋄ Takahashi RCA[5]	19	35	67	131	259	515	1027
⋆ Takahashi Combine Adder[5]	216	270	324	378	432	486	540
⋄ Gidney RCA[6]	16	32	64	128	256	512	1024
⋆ Our Adder	59	71	83	95	107	119	131
• Our Adder	56	68	80	92	104	116	128
⋄ Our Adder	25	43	55	67	79	91	103

(c) QC Comparison

Structure \ input size	16	32	64	128	256	512	1024
⋆ VBE RCA[1]	49	97	193	385	769	1537	3073
⋄ VBE RCA[1]	49	97	193	385	769	1537	3073
⋆ Cuccaro RCA[2]	34	66	130	258	514	1026	2050
⋄ Cuccaro RCA[2]	34	66	130	258	514	1026	2050
⋆ Draper In-place CLA[3]	59	122	249	504	1015	2038	4085
⋄ Draper In-place CLA[3]	59	122	249	504	1015	2038	4085
⋆ Takahashi Adder[4]	44	83	160	310	608	1194	2355
⋆ Takahashi RCA[5]	33	65	129	257	513	1025	2049
⋄ Takahashi RCA[5]	33	65	129	257	513	1025	2049
⋆ Takahashi Combine Adder[5]	44	83	160	310	608	1194	2355
⋄ Gidney RCA[6]	47	95	191	383	767	1535	3071
⋆ Our Adder	54	116	243	503	1025	2067	4150
• Our Adder	54	116	243	503	1025	2067	4150
⋄ Our Adder	60	134	287	584	1181	2373	4762

Table 2. Compare the cost required by Draper's out-of-place CLAs and the higher radix adders.
The data of the comparison between Draper's out-of-place adder and our simplified adder are shown in the table below.

(a) T-count Comparison

Structure \ Input size	16	32	64	128	256	512	1024
⋆ Draper Out-of-place CLA[3]	448	987	2086	4305	8764	17703	35602
⋄ Draper Out-of-place CLA[3]	338	727	1516	3105	6294	12683	25472
⋆ Our Adder	308	644	1316	2660	5348	10724	21476
• Our Adder	266	554	1130	2282	4586	9194	18410
⋄ Our Adder	178	362	730	1466	2938	5882	11770

(b) T-depth Comparison

Structure \ Input size	16	32	64	128	256	512	1024
⋆ Draper Out-of-place CLA[3]	30	36	42	48	54	60	66
⋄ Draper Out-of-place CLA[3]	25	31	37	43	49	55	61
⋆ Our Adder	35	41	47	53	59	65	71
• Our Adder	38	44	50	56	62	68	74
⋄ Our Adder	28	34	40	46	52	58	64

(c) QC Comparison

Structure \ Input size	16	32	64	128	256	512	1024
⋆ Draper Out-of-place CLA[3]	60	123	250	505	1016	2039	4086
⋄ Draper Out-of-place CLA[3]	60	123	250	505	1016	2039	4086
⋆ Our Adder	54	116	243	503	1025	2067	4150
• Our Adder	54	116	243	503	1025	2067	4150
⋄ Our Adder	60	134	287	584	1181	2373	4762
